# Clomiphene Citrate versus Recombinant FSH in intrauterine insemination cycles with mono- or bi-follicular development

**DOI:** 10.5935/1518-0557.20200106

**Published:** 2021

**Authors:** Vehbi Yavuz Tokgoz, Yavuz Emre Sukur, Batuhan Ozmen, Murat Sonmezer, Bulent Berker, Rusen Aytac, Cem Somer Atabekoglu

**Affiliations:** 1 Department of Obstetrics and Gynecology, Eskisehir Osmangazi University Faculty of Medicine, Eskisehir, Turkey; 2 Department of Obstetrics and Gynecology, Ankara University Faculty of Medicine, Ankara, Turkey

**Keywords:** intrauterine insemination, follicle stimulating hormone (FSH), ovarian stimulation, clomiphene citrate, mono-follicular, bi-follicular

## Abstract

**Objective:**

The present study aims to assess the success of controlled ovarian stimulation in intrauterine insemination cycles stimulated by recombinant-FSH and Clomiphene citrate for either mono- or bi-follicular development.

**Methods:**

We assessed 870 infertile patients treated with controlled ovarian stimulation in intrauterine insemination cycles at a university-based infertility clinic between January 2012 and December 2017. We compared the cycles stimulated by clomiphene citrate and recombinant-FSH in two set-ups; mono- and bi-follicular development. The main outcome measure was the clinical pregnancy rate per cycle.

**Results:**

The demographic and cycle parameters were similar between the groups, except for endometrial thickness on the day of hCG administration, which was higher in the recombinant-FSH group than the clomiphene citrate group. The overall clinical pregnancy rates in clomiphene citrate and recombinant-FSH groups were 9.8% and 10.3%, respectively (*p*=0.940). Regarding the entire cohort, clinical pregnancy was significantly higher in cases of bi-follicular development when compared to mono-follicular development (16.8% vs. 10.2%, respectively; *p*=0.009).

**Conclusions:**

Clomiphene citrate and recombinant-FSH have similar success rates in terms of clinical pregnancy, in either mono-follicular development or bi-follicular development. Clomiphene citrate and recombinant-FSH cycles resulted in comparable rates of bi-follicular development, which significantly increases clinical pregnancy rate. Clomiphene citrate and recombinant-FSH have similar success rates in terms of clinical pregnancy, in either mono-follicular development or bi-follicular development.

## INTRODUCTION

Controlled ovarian stimulation (COS) combined with intrauterine insemination (IUI) is a common fertility treatment, approved as a first-line treatment option for unexplained infertility, mild male factor infertility and minimal-mild endometriosis (Cohlen *et al*., 1998; Kennedy *et al*., 2005; Veltman-Verhulst *et al*., 2016). Clomiphene citrate (CC) is mainly accepted as the first-line drug in COS-IUI cycles, due to its feasibility and cost-effectiveness (Berker *et al*., 2011; Dankert *et al*., 2007). However, a meta-analysis showed that FSH (follicle-stimulating hormone) is superior to CC in terms of clinical pregnancy rates (Cantineau *et al*., 2007). Although the primary aim is to achieve a monofollicular growth in COS-IUI cycles, previous studies suggested significantly higher pregnancy rates with multifollicular development (Berker *et al*. 2011; van Rumste *et al*., 2008). Another important concern is the relatively high risk of multiple pregnancy rates in cases of multifollicular growth (Berkovitz *et al.*, 2017; Garrido et al., 2007). However, several studies failed to show a significant difference between one and two preovulatory follicles in terms of multiple pregnancy rates (Dickey *et al*. 2001; van Rumste *et al*., 2008). In a recent randomized controlled trial (RCT), we showed that COS-IUI with FSH resulted in a higher number of preovulatory follicles, when compared to COS-IUI with CC (Berker *et al*., 2011).

The superiority of FSH regarding pregnancy rates seems to be due to increased multifollicular growth. However, there is still a gap in the literature regarding the success of FSH and CC when an equal number of follicles develop. This study aimed to compare the pregnancy rates of COS-IUI cycles utilizing FSH and CC in the case of both mono-follicular and bi-follicular development.

## MATERIALS AND METHODS

In this retrospective cohort study, we assessed infertile patients treated with COS-IUI at a university-based infertility clinic between January 2012 and December 2017. The Institutional Review Board of Ankara University approved the study (IRB approval no. and date: 31043 - 26/06/2018). The inclusion criteria were females, aged <38 years, failure to conceive for ≥12 months of unprotected sexual intercourse, with at least one patent fallopian tube confirmed by hysterosalpingography (HSG) or laparoscopy, basal FSH levels <12 IU/l. We have also determined the causes of infertility, as female factor, mild male factor and unexplained infertility. Semen samples compatible with the criteria from the World Health Organization (WHO) were determined as normal (WHO, 2010). We stated the mild male factor as failing to meet the WHO criteria, but having total progressive motile sperm count (TMSC) > 5 million. The exclusion criteria were the growth of at least 3 follicles >17 mm, bilateral tubal occlusion, presence of ovarian cysts larger than 15 mm on baseline ultrasound examination, severe endometriosis (according to the revised American Fertility Society stage III and IV), and severe male factor infertility (the total number of inseminated motile sperm count <1 million). All couples had a standard infertility work-up including medical history, physical examination, semen analysis (abnormal semen test results confirmed by a second analysis 6 weeks later), HSG to confirm tubal patency, and basal hormone profile. We also included the cycles that had polycystic ovary syndrome (PCOS) or oligo-ovulatory menstrual profile.

A baseline ultrasound exam was performed on day 2 or 3 before starting a cycle. Clomiphene citrate (Klomen; Kocak Farma, Istanbul, Turkey) was administered at a 100 mg daily dose for five days, starting from cycle days 3-5. Recombinant FSH (Gonal-F; Merck-Serono, Istanbul, Turkey, or Puregon; Organon, Istanbul, Turkey) was administered according to body mass index (BMI). Starting rFSH dose was 75 IU/day for women with BMI <25 kg/m2 and 100 IU/day for women with BMI ≥25 kg/m2. rFSH injections were commenced on cycle day 2-4. The first monitoring scan was performed on the 6th day of gonadotrophin stimulation, and repeated after 2 or 3 days, depending on the follicular growth. HCG (Pregnyl; Schering-Plough, Istanbul, Turkey) 10,000 IU was administered when the leading follicle reached ≥18 mm in both groups. A single IUI was performed 36-40 h after hCG injection.

Semen samples were taken by a masturbation and collected in sterile containers. Semen samples used for insemination were processed within one hour after ejaculation, using a density gradient centrifugation followed by washing with culture medium. The women had bed rest for 15-20 min after the IUI.

The first study group consisted of patients stimulated by CC and the second study group consisted of patients stimulated by rFSH. The distribution of the type of stimulations with CC and rFSH were not different within years, and approximately 25% and 75% for CC and rFSH, respectively. The success rates of the groups were compared under two separate conditions in cases of mono-follicular development or case of bi-follicular development. Bi-follicular development was defined as two dominant follicles reaching a minimum of 17 mm in diameter. Biochemical pregnancy was defined by positive serum β-hCG levels 2 weeks after IUI. Clinical pregnancy was defined as the presence of a heartbeat at 7-8^th^ gestational weeks. The primary outcome measurement was the clinical pregnancy rate (CPR).

### Statistical Analysis

We analyzed the data using the SPSS Version 21.0 (IBM Corporation, Armonk, NYC, USA). The samples were tested with the Kolmogorov-Smirnov test to determine the normality of distributions. Continuous variables were compared with the Student’s t-test (for normally distributed data) and the Mann-Whitney U test (for skewed data). Categorical variables were compared with the Chi-square test. We used the multivariable logistic regression analysis with a model-building strategy to determine independent predictors of clinical pregnancy in IUI cycles. A *p-*value of <0.05 was considered statistically significant.

## RESULTS

**Figure 1:**
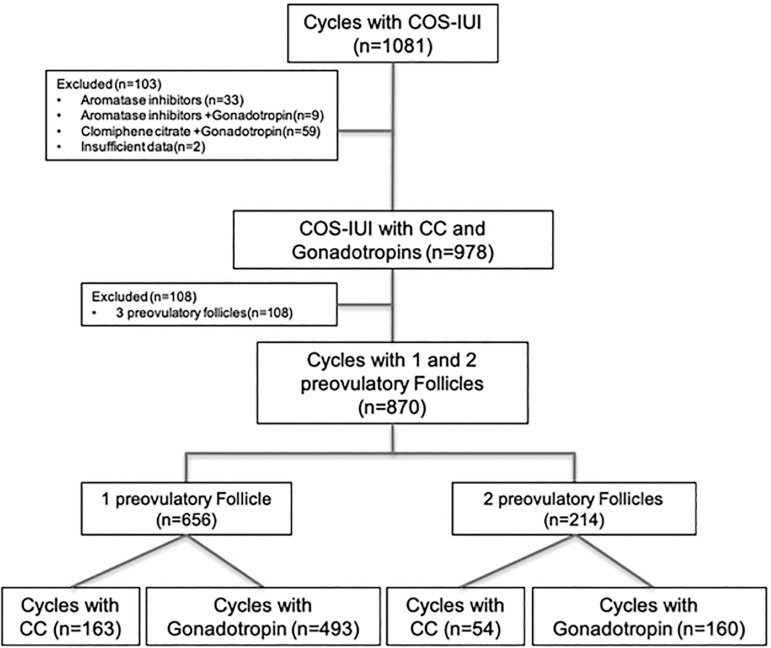
Flowchart of the study population.

A total of 1081 womens’ cycles were assessed for eligibility. The cycles stimulated with other than CC or rFSH were excluded, as well as the cycles in which three or more follicles developed (Figure 1). Only first IUI cycles were included to prevent crossover biases. As a result, the final analyses were performed with 870 cycles. Table 1 represents the demographic parameters, cycle characteristics and pregnancy rates of the study groups. The groups were mainly similar to each other. The female age and the duration of infertility were significantly higher in the rFSH group than in the CC group (*p*=0.003 and *p*=0.009, respectively). As expected, endometrial thickness on the day of hCG was significantly lower in the CC group than in the rFSH group, due to the anti-estrogenic effect of CC (7.77±2.12 vs. 9.73±2.33, respectively; *p*=0.001). The etiologies of infertility, basal antral follicle count (AFC) and TMSC were significantly different between the CC and rFSH groups. We did not find any impact of these differences on pregnancy outcomes, although AFC and TMSC were higher in the CC group than in the rFSH group.

**Table 1:** Demographics and cycle characteristics of the study population.

	CC cycles (n=217)	rFSH cycles (n=653)	*p* value
Age (years) ^†^	28.3±6.4	29.2±4.9	0.003
BMI (kg/m2) ^†^	28.3±2.8	25.1±0.5	0.136
Baseline FSH (mIU/mL) ^†^	6.7±2.2	6.9±4.2	0.744
Baseline LH (mIU/mL) ^†^	5.7±4.0	5.5±3.4	0.697
Baseline E2 (pg/mL) ^†^	51.8±3.1	56.2±2.2	0.054
Duration of infertility (years) †	4.0±3.0	5.6±3.7	0.009
Secondary infertility, n (%)	57 (25.9)	70 (10.7)	0.001
Cause, n (%) - Female factor - Mild male factor - Unexplained	22 (10.2) 32 (14.7) 163 (75.1)	82 (12.5) 50 (7.7) 521 (79.8)	0.009
Antral follicle count(number) ^†^	16.3±0.7	13.3±0.5	0.005
Endometrial thickness on hCG day (mm) ^†^	7.8±2.1	9.7±2.3	0.001
TMSC (x10^6^) ^†^	118.0±9.4	88.5±4.7	0.011
IMC (x10^6^) ^†^	52.6±4.5	37.6±1.8	0.104
Number of intermediate follicles (14-16 mm)	0.50±0.27	0.76±0.16	0.021
Clinical Pregnancy rate (%)	12.0	11.8	0.512

† Data presented as mean±SD

SD: standard deviation; BMI: body mass index; TPMS: total motile sperm count; IMC: inseminated motile count.

We also recorded the number of accompanying intermediate follicles (14-16 mm), and found significant tendencies in favor of the rFSH group. The overall clinical pregnancy rates in the CC and rFSH groups were 11.8% and 12.0%, respectively (*p*=0.512). Regarding the entire cohort, the CPR was significantly higher in the case of bi-follicular development when compared to mono-follicular development (16.8% vs. 10.2%, respectively; *p*=0.009). There were no significant differences regarding to the number of intermediate follicles between the mono- and the bi-follicular growth groups, although we found higher intermediate follicles in the cycles that had bi-follicular growth (0.67 vs. 0.77, *p*=0.596). Multiple pregnancy rates were similar between mono- and bi-follicular developments (2% vs. 4%, respectively). We only had twin pregnancies in our study, there were no triplets or other multiple pregnancies. 

**Figure 2:**
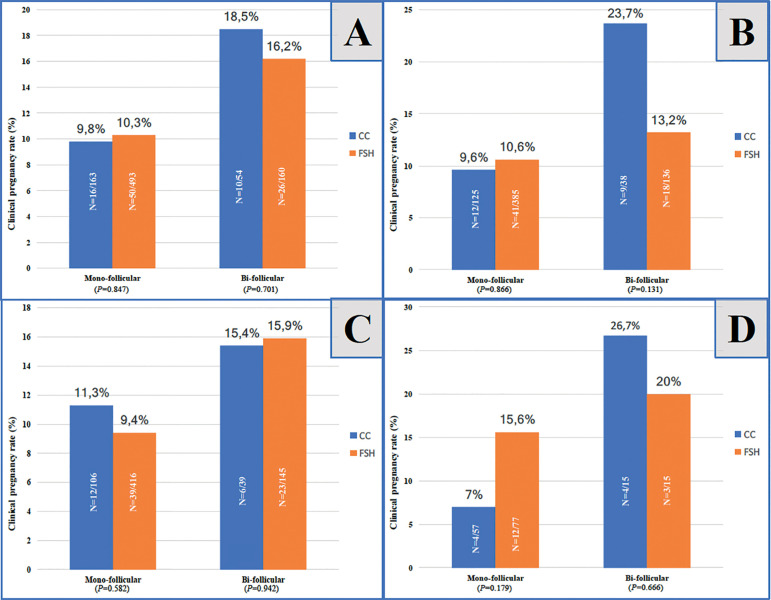
Clinical pregnancy rates regarding stimulation and no. of follicles developed (2A: overall, 2B: unexplained infertility, 2C: Non-PCOS reasons, 2D: PCOS).

Figure 2 shows the CPR for each group under the conditions of mono- and bi-follicular growth. The overall CPR was similar in both mono- and bi-follicular developments between the groups (Figure 2A). In addition, according to the cause of infertility, the subgroup analyses showed no statistically significant differences (Figures 2B-2D). The subgroups were unexplained infertility, PCOS and non-PCOS cases. The rFSH group had higher clinical pregnancy rates in the cycles that had PCOS and mono-follicular growth; on the other hand CC was superior to rFSH in the bi-follicular growth for PCOS (Figure 2D). There was a huge difference between the CC and the rFSH groups in favor of CC in the subgroup of unexplained infertility, although it was not statistically significant (Figure 2B). We thought that the small number of cases might explain that non-significant difference.

A multi-variable analysis was performed to identify the factors, which have an impact on CPR. We considered the parameters that were significantly different between the CC and the FSH group. Female age, infertility duration, antral follicle count, TPMS (total motile sperm count), IMC (inseminated motile count), type of stimulation, endometrial thickness on the hCG day, number of intermediate follicles and bi-follicular development were analyzed on the logistic regression analysis. Backward stepwise logistic regression was performed and then, bi-follicular development and endometrial thickness on the day of hCG significantly increased the odds of clinical pregnancy (Table 2). However, no other factors had a significant influence on the success rate of an IUI cycle.

**Table 2:** Multivariate logistic regression analysis demonstrating predictors of clinical pregnancies in OS-IUI cycles.

	OR	95% Confidence Interval	*p* value
Bi-follicular growth	2.556	1.234 - 5.292	0.011
Endometrial thickness	1.210	1.055 - 1.387	0.006

## DISCUSSION

In the present study, we identified that the CPR was similar between IUI cycles stimulated by CC or rFSH, in cases of mono- and bi-follicular development, separately. We also determined that bi-follicular development and increment in endometrial thickness increased the likelihood of clinical pregnancy in COS-IUI.

The impact of multi-follicular development on the pregnancy rates in COS-IUI cycles has been investigated for many years and contradictive results have been achieved. Tomlinson *et al.* (1996) identified the most significant prognostic factors for IUI cycles as the number of follicles, endometrial thickness, duration of infertility, and progressive motility of semen. They determined the chance of conceiving as 7.6% and 26% when one or two preovulatory follicles developed, respectively. In another retrospective analysis, CPR was calculated for different follicle numbers and estradiol levels on the day of hCG in gonadotropin-induced IUI cycles (Ozcakir *et al*., 2002). The CPR for one, two, three, and four or more preovulatory follicles were 14.2%, 14.2%, 27.5%, and 24%, respectively. Although not statistically significant, the CPR was increased with three or more follicles ≥18 mm on the day of hCG. The authors failed to show a significant difference between one or two preovulatory follicles in terms of CPR (Ozcakir *et al*., 2002). According to the findings of a meta-analysis, the pooled OR (odds ratio) of CPR after two, three and four dominant follicles development were 1.59, 2.02 and 2.04, respectively, when compared to mono-follicular development (*p*<0.05) (van Rumste *et al*., 2008). However, the follicle sizes on the day of hCG were not homogeneous (van Rumste *et al*., 2008). Evans *et al.* (2020) recently concluded that two preovulatory follicles improved the pregnancy rates, but more than two follicles did not contribute the chance of singleton pregnancy. We excluded the cycles that had more than two preovulatory follicles (>17 mm) and found that bi-follicular growth significantly increased the clinical pregnancy rate. 

In a recent study by Merviel *et al*. (2010), they concluded that the number of follicles >16 mm and estradiol concentration on the day of triggering significantly improved pregnancy outcomes. Moreover, in a previous RCT, we already demonstrated that multi-follicular development was higher in rFSH-IUI than CC-IUI (Berker *et al*., 2011). Although non-significant, the CPR was higher in multi-follicular development than mono-follicular development in both CC-IUI and rFSH-IUI cycles (Berker *et al*., 2011). Evans *et al.* (2020) defined the mature follicles as a follicle size above 14 mm, and they found increasing pregnancy rates with increased number of mature follicles. Moreover, they showed higher multiple gestations with this increase. We defined the preovulatory follicle as 17 mm and above, additionally we did not find significant beneficial effects of accompanying intermediate follicles in the study groups. In the present study, we also found higher CPR with two preovulatory follicles development when compared to mono-follicular development (*p*=0.009). Furthermore, we performed logistic regression analyses, which showed bi-follicular development as one of the two significant predictors of success in COS-IUI cycles (OR: ~ 2.5).

Female age is one of the most important predictors in COS-IUI cycles, and it has been speculated that female age negatively affected the reproductive outcomes. Merviel *et al.* (2010) found that the women under 30 had higher pregnancy rates compared to those of advanced age. In a previous study, we did not find any significant effect of female age in the first COS-IUI cycles of patients (Berker *et al*., 2011). In a very recent study, Immediata *et al.* (2020) concluded that female age and FSH levels significantly influenced the pregnancy outcomes in COS-IUI cycles. The duration of infertility is another factor that is associated with the clinical outcomes. Aydin *et al.* (2013) reported that the duration of infertility was higher in the non-pregnant group, but not significantly. We established that the female age and duration of infertility were significantly lower in the CC group, whereas these significant differences were not reflected on the clinical pregnancy rate.

Several studies have compared CC and FSH in IUI cycles, and most suggested superiority of FSH to CC in terms of CPR (Berker *et al*. 2011; Peeraer *et al*., 2015; Shalom-Paz et al. 2014). In a multicenter RCT, Peeraer *et al.* (2015) found that CPR was significantly higher in hMG (human menopausal gonadotrophin)-stimulated IUI cycles than CC-IUI cycles although the number of preovulatory follicles >14 mm was significantly lower in the hMG group than in the CC group (13% vs. 7.1%, *p*=0.02; RR:1.8). The authors explained this incompatibility with the lower dose of gonadotrophins in their study. On the contrary, Dankert *et al.* (2007) found comparable pregnancy rates between CC and gonadotrophin, and showed a tendency in favor of the CC-treatment arm. They also reported that the number of preovulatory follicles higher than 14 mm did not influence pregnancy outcomes. In a recent study, Danhof *et al.* (2019) questioned whether it is possible to get better pregnancy outcomes with FSH instead of CC in intrauterine insemination cycles for unexplained infertility. The authors reported that neither FSH nor CC had a significant superiority to the other agent. They also showed that the number of follicles above 14 mm were similar between the FSH and the CC groups (1.8 vs. 1.9). We found that the CPR was higher in bi-follicular growth than mono-follicular growth, similar to the literature. However, we failed to show a significant difference between rFSH and CC when adjusted for the number of follicles developed. In the literature, the CPR was generally higher in the FSH groups when the multi-follicular growth occurs, but the differences were not statistically significant (Berker *et al*., 2011; Huang *et al*., 2018). Zolton *et al.* (2020) also justified the effect of gonadotrophins against the oral agents for unexplained infertility. In this meta-analysis, they established that the clinical pregnancy and live birth rates were not significantly higher with the use of gonadotrophins for intrauterine insemination cycles in unexplained infertility, but they obtained an increased relative risk of multiple pregnancy with gonadotrophins.

In general, it has been thought that multi-follicular growth was significantly higher in FSH cycles than in CC cycles. In our previous study, we observed increased multi-follicular growth in the FSH group without a significant impact on CPR (Berker *et al*., 2011). On the contrary, several studies reported higher multi-follicular growth on the day of hCG in CC cycles compared to FSH cycles (Danhof *et al*., 2018; Peeraer *et al*., 2015). However, Huang *et al.* (2018) reported that the percentages of mono-follicular development were 67% and 72.9% for the CC and FSH groups, respectively. In the present study, we found similar mono-follicular development rates; 75.1% and 75.5% in the CC and FSH treatment arms, respectively. The rFSH groups had women who were of older and had a longer duration of infertility compared to the CC group, and it might make a disadvantage to achieve more improved cycle outcomes for the rFSH group of patients. On the other hand, the finding of similar mono-follicular development rates may support the hypothesis of similar success rates between two types of treatments, as the CPR was comparable in the subgroups of mono- and bi-follicular development.

Huang *et al.* (2018) analyzed mono- and multi-follicular growth separately, and observed similar pregnancy outcomes between CC, letrozole, and gonadotrophin in infertile women with PCOS undergoing IUI. Although non-significant, the CPR was higher in multi-follicular growth than mono-follicular growth (19.1% vs. 16.1%, respectively). In the mono- and multi-follicular subgroup analyses, similar CPR were determined for both CC and gonadotrophins (16.4% vs. 16.1% and 20% vs. 21.7%, respectively). They concluded that similar live birth rates were achieved with letrozole, CC or gonadotrophins; moreover, multi-follicular growth did not improve pregnancy outcomes but it increased the multiple pregnancy rates (OR=22.4) (Huang *et al*., 2018). In addition, a recent RCT by Danhof *et al.* (2018) also, failed to show significant differences between FSH and CC in couples with unexplained subfertility undergoing IUI. The cumulative CPR was 32% and 27% for FSH and CC, respectively, and also the mean numbers of follicles ≥14 mm were similar (1.8 vs. 1.9, respectively). However, they did not perform a subgroup analysis for mono- or multi-follicular growth regarding the effectiveness of FSH and CC. In the present study, there was a higher CPR in the bi-follicular growth compared with the mono-follicular growth, and we also performed a comparative analysis to detect the effectiveness of rFSH and CC in mono- and bi-follicular developments separately. Bahadur *et al.* (2017) also concluded that pregnancy rates may be improved in IUI cycles using gonadotrophin with 2 preovulatory follicles compared to CC cycles. In another opinion article, Bahadur & Homburg (2019) suggested that the evidence about IUI should be updated. They also concluded that IUI was to be efficient with low multiple pregnancy rates, reasonable live birth rates and cost-effectivity, when compared with the IVF treatment. Few studies investigate the pregnancy outcomes between one and two preovulatory follicles development in IUI cycles. From this point of view, we have performed the analysis to detect differences. We did not show any significant differences between rFSH and CC for unexplained, non-PCOS, and PCOS subgroups in terms of mono- or bi-follicular development, despite the CC group having lower age and shorter infertility duration.

The cost-effectiveness analysis showed controversial results about using gonadotrophins or CC in intrauterine insemination cycles. Peeraer *et al.* (2018) suggested that low-dose gonadotrophin was more expensive than CC, whereas it was more cost-effective to achieve clinical pregnancy compared to the stimulation with CC. On the other hand, in a very recent study, Danhof *et al*. (2020) concluded that gonadotrophins, a more expensive agent, did not show significant benefits on pregnancy outcomes when compared to CC, even when used under strict cancellation criteria.

We showed non-significant higher multiple gestation rates in the bi-follicular development group as similar to the literature. Evans *et al*. (2020) reported that multiple pregnancy rates increased with more mature follicles, but they concluded that more than two mature follicles significantly increased the multiple gestations compared to only two mature follicles.

Despite the retrospective nature of the analyses and heterogeneity between the groups in terms of age, antral follicle count, and TMSC, that we recognize as limitations of our study due to unequal groups compromising the rFSH cohort, the systematic exploration of individual parameters, large sample size in each group, and the multivariate approach add credence to our observations. We focused on evaluating the CPR among the study population so we did not observe the LBR. It may be another limitation of our study. Endometrial thickness was significantly higher in the rFSH group than in the CC group. However, this was not reflected on the CPR outcome, possibly due to higher age, lower antral follicle count, and lower TMSC values in the rFSH group.

This clinical study found that the development of two preovulatory follicles increased the clinical pregnancy rate compared to mono-follicular development in intrauterine insemination cycles. However, gonadotropin did not have a significantly higher pregnancy rate than clomiphene citrate in both mono- and bi-follicular growth. We identified the importance of two preovulatory follicles development on the clinical pregnancy rate, without increasing the multiple pregnancies.

## CONCLUSION

In the present study; bi-follicular development has a significant impact on IUI cycles, and CC and rFSH cycles result in comparable rates of bi-follicular development. In any indication, CC and FSH have similar success rates in terms of CPR, in either mono-follicular development or bi-follicular development due to the compromised rFSH cohort having higher age and longer duration of infertility.
